# Establishing a Clinical Brain-Computer Interface Program for Children With Severe Neurological Disabilities

**DOI:** 10.7759/cureus.26215

**Published:** 2022-06-22

**Authors:** Zeanna Jadavji, Ephrem Zewdie, Dion Kelly, Eli Kinney-Lang, Ion Robu, Adam Kirton

**Affiliations:** 1 Clinical Neuroscience, University of Calgary, Calgary, CAN; 2 Physical Medicine and Rehabilitation, Alberta Children's Hospital, Calgary, CAN; 3 Pediatrics, Clinical Neuroscience, University of Calgary, Calgary, CAN

**Keywords:** brain computer interface, cerebral palsy types, non-verbal communication, electroencephalography (eeg), perinatal stroke, neuro-technology, clinical neuroscience

## Abstract

Background: Children with severe motor impairment but intact cognition are deprived of fundamental human rights. Quadriplegic cerebral palsy is the most common scenario where rehabilitation options remain limited. Brain-computer interfaces (BCI) represent a potential solution, but pediatric populations have been neglected. Direct engagement of children and families could provide meaningful opportunities while informing program development. We describe a patient-centered, clinical, non-invasive pediatric BCI program.

Methods: Eligible children were identified within a population-based, tertiary care children’s hospital. Criteria included 1) age six to 18 years, 2) severe physical disability (non-ambulatory, minimal hand use), 3) severely limited speech, and 4) evidence of grade 1 cognitive capacity. After initial screening for BCI competency, participants attended regular sessions, attempting commercially available and customized systems to play computer games, control devices, and attempt communication.

Results: We report the first 10 participants (median 11 years, range 6-16, 60% male). Over 334 hours of participation, there were no serious adverse events. BCI training was well tolerated, with favorable feedback from children and parents. All but one participant demonstrated the ability to perform BCI tasks. The majority performed well, using motor imagery based tasks for games and entertainment. Difficulties were most significant using P300, visual evoked potential based paradigms where maintenance of attention was challenging. Children and families expressed interest in continuing and informing program development.

Conclusions: Patient-centered clinical BCI programs are feasible for children with severe disabilities. Carefully selected participants can often learn quickly to perform meaningful tasks on readily available systems. Patient and family motivation and engagement appear high.

## Introduction

Cerebral palsy (CP) causes lifelong disability for millions and is the most common pediatric motor disability [1]. Children suffering from quadriplegic CP are often unable to achieve self-directed ambulation, manual control, or communication [2,3]. Functional independence is strongly correlated to quality of life for children with CP but is often virtually absent for those with quadriplegic CP [4,5].

Children with severe physical disabilities may have sparing of cognitive function and can be intellectually typical. This tragic condition where a capable young person is trapped inside a body that cannot move or communicate is analogous to locked-in syndrome [6]. Being fully aware and capable yet unable to interact with the world deprives such individuals of inalienable, fundamental human rights. The United Nations Convention on the Rights of Persons with Disabilities (CRPD) makes particular reference to the plight of disabled children where quadriplegic CP provides a compelling example [7].

Brain-computer interface (BCI) technology can afford new avenues of independence and interaction for such individuals. BCIs translate intention-related electrical activity recorded from the user’s brain into computer commands to control external devices [8]. Advancements in non-invasive signal acquisition provide an opportunity for BCI control through surface electroencephalography (EEG). A study of 12 adults with locked-in syndrome suggested that patients could achieve high classification accuracies and simple yes/no communication using non-invasive BCI [9]. We recently demonstrated that clinician awareness of BCI technology is poor despite high estimates of clinical utility [10].

Pediatric populations have been neglected in BCI research despite many children with severe disability suffering lifelong morbidity. However, we have shown that both typically developing school-aged children and those with perinatal brain injury can successfully operate simple BCI systems [11,12]. A fundamental gap exists between rapidly progressing technologies and the patients that could benefit. Clinical research may provide opportunities for affected children to try emerging BCI systems while engaging families to inform program development [13]. We describe early results from our attempt to develop a patient-centered, non-invasive clinical BCI program for children with severe neurological disabilities.

## Materials and methods

Participants

Population-based screening of potentially eligible participants was performed in Southern Alberta, Canada (population approx. 2.2 million). All health care professionals at the single referral tertiary care academic children’s hospital caring for severely disabled children with relevant expertise (neurologists, physiatrists, therapists, etc) were contacted. They were asked to identify children with the following specific criteria: (1) age six to 18 years, (2) severe physical disability including non-ambulatory (GMFCS V) and minimal hand use (MACS 5), (3) non-verbal or severely limited expressive speech, and (4) evidence from parents, teachers, and/or therapists of grade 1 level cognitive capacity or better. A single invitation to refer was distributed in October 2017, with open accrual encouraged between then and the time of this analysis (March 2020).

Referrals were reviewed by the study neurologist. Clinical information was obtained from the medical record and discussion with clinicians familiar with the child and family. This information included confirmation of the underlying disorder, CP classification, and information on hearing and vision status, cognitive potential, comorbid conditions including epilepsy, use of different assistive technology and communication strategies, and family dynamics. Neuroimaging was reviewed by the study neurologist to confirm diagnosis, absence of additional neuropathology, and presence of fundamental neuroanatomy consistent with the reported level of cognitive potential and potential for EEG-based BCI applications (i.e., substantial preservation of cerebral cortex in at least one hemisphere).

A database of potential participants was maintained in estimated rank order of potential success based on the above criteria and practical, patient-centered factors including, ability to attend regular appointments. Intake proceeded down this list as the growing BCI program could accommodate. Parents or guardians provided written informed consent. Methods were approved by the University of Calgary Research Ethics Board REB15-2567.

Program structure

Participants were seen in clinic by the study neurologist to acquire relevant history, physical examination and to explain the basic concept of BCI. To maintain realistic expectations, the program was described as exploratory with no guaranteed success and assurance of no impact on ongoing standard clinical care.

This was followed by an introductory BCI session to gauge the child’s potential ability to control a simple BCI. Initial sessions were completed using the EPOC+ where participants attempted simple tasks as outlined below. If they demonstrated some ability to use the BCI with at least partial control, they were invited to join the BCI program. If not, options were provided to 1) try the same or new systems on a subsequent day, 2) be notified to return when new technologies became available.

After enrollment, regular BCI sessions were offered to the families. Sessions were scheduled for an hour and adjusted according to the child’s engagement, interests, and ability. New activities were introduced gradually, and their use was adjusted according to the same factors.

BCI systems

**Figure 1:**
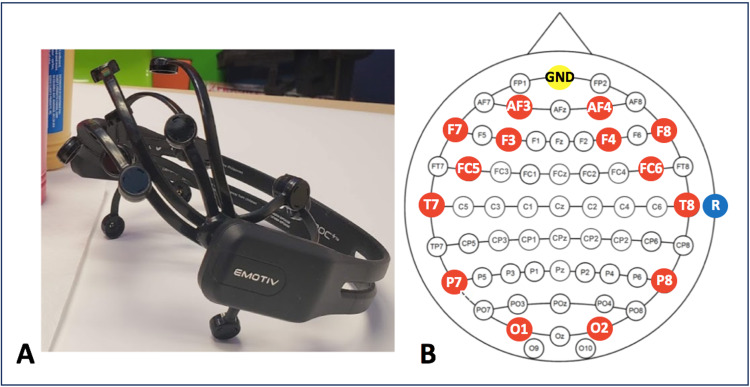
BCI systems. (A) The EMOTIV EPOC+ headset (B) The EMOTIV EPOC+ EEG montage. Red circles indicate EEG recording channels. Yellow and blue circles represent ground and reference electrodes, respectively.

**Figure 2:**
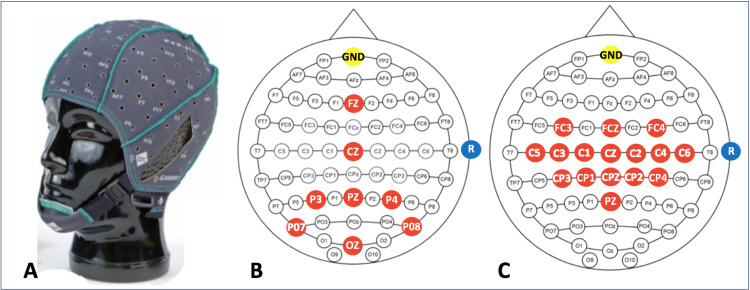
BCI systems continued. (A) The g.tec intendix and mindBEAGLE headset. The EEG headset is sizeable to individual participants (B) The g.tec intendix montage (C) The g.tec mindBEAGLE montage. Red circles indicate EEG recording channels. Yellow and blue circles represent ground and reference electrodes, respectively.

The technical and human resources of the BCI program are summarized in Table 1. The BCI program has incorporated the use of three commercially available systems: 1) EMOTIV EPOC+ (EMOTIV, San Francisco, CA, USA), 2) g.tec intendiX (g.tec, Schiedleberg, Austria), and 3) g.tec mindBEAGLE (g.tec). Headset design and system details are included in Figures 1, 2 and Table 2, respectively. 

**Table 1:** BCI Program Team, Structure and Resources. Personnel and associated roles of team members involved in the brain-computer interface (BCI) program.

Human Resources	Credentials	Roles
Patients and families	Lived experience	BCI end users attempting different tasks and providing feedback
Pediatric Neurologist	MD, expert in perinatal brain injury Professor, clinician-scientist	Identifying eligible participants, maintaining recruitment database, consulting on relevant clinical information
Lead Biomedical Engineer	PhD BME, expert in neuro-technology	Design adaptation and implementation of BCI technology
Augmented communication specialist	Certified Occupational Therapist	Consulting on patient seating, screen positioning and alternate assistive technology
BCI engineer	MSc BME	Technology design and optimization
BCI engineer	PhD, now PDF	Technology design and optimization
Clinical researcher	BSc, now MD/PhD candidate	Design and implementation of BCI program and patient engagement
Clinical researcher	MBT, now PhD candidate	Assisting in BCI sessions
Rehabilitation Engineer	MSc, Biomedical Equipment Technologist	Technology design
Referring specialists	10-12 neurologists, physiatrists, therapists	Identifying eligible participants
Summer students	N=5-6 per year	Condensed research projects
Study coordinator		Administrative responsibilities

**Table 2:** BCI Systems. Description of each system used in the pediatric brain-computer interface (BCI) program with specifics on EEG data collection and sampling as well as paradigms used. Cost estimates of technical resources included. MI: Motor Imagery, VEP: Visual Evoked Potential, AEP: Auditory Evoked Potential, VTP: Vibrotactile Evoked Potential

BCI System	Conductive solution	International EEG System	Recording Electrode Configuration		Sampling Rate	Filtering	Associated Paradigms
EMOTIV EPOC+	Saline	10-20	14 channel AF3, AF4, F3, F4, F7, F8, FC5, FC6, P7, P8, T7, T8, O1, O2		128Hz	Bandpass filter: 0.16-45Hz	MI
g.tec intendiX	Gel	10-10	8 channel Fz, Cz, P3, Pz, P4, P07, Oz,P08		256Hz	Notch filter: 60 Hz	VEP
g.tec mindBEAGLE	Gel	10-10	16 channel FC3,FCz,FC4,C5,C3, C1,Cz,C2,C4,C6,CP3, CP1,CPz,CP2,CP4 and Pz		256Hz	Notch filter: 60 Hz	MI, AEP, VTP
Technological Resources		Estimated Cost		Roles			
EMOTIV EPOC+		$699.00 USD		Wireless EEG headset and software			
g.tec intendiX		$15-20K USD		BCI suite for P300 spelling			
g.tec mindBEAGLE		$30-35K USD		BCI suite for assessment of consciousness and communication			
Sphero SPRK+		$129 USD		Used in maze and painting activities			
Remote controlled (RC) toy car		$20 USD		Controlled by BCI			
Arduino (board + starter kit)		$135 USD		Interfacing with RC toy car			

Different BCI paradigms were used for device control. Motor imagery was performed by imagining an intended movement without actual movement or muscle activation. Motor imagery causes EEG amplitude changes over the sensorimotor cortex that the computer recognizes and translates into a keyboard output for device control [14]. External stimulation paradigms were based on visual, auditory, and vibrotactile evoked potentials measured at 300ms latency (P300) [15]. These three modes make use of the well-defined oddball paradigm where participants were asked to attend to specific, target stimuli while ignoring non-target stimuli [16-18].

Activities and training

Motor imagery was used with the EPOC+ to control computer-based games, drive a toy car, control a Sphero SPRK+ robot (Sphero, Boulder, CO, USA) and select items on an iPad. Training across all EPOC+ tasks included brief 8-second windows of training where participants practiced being relaxed and then imagined “pushing” the item they were attempting to control. During “pushing”, whenever the power of the EEG frequency spectrum passed a preset threshold, a keyboard output was produced. If able, patients could raise their eyebrows to generate an electromyography (EMG) signal that was mapped to an additional keyboard output. During the mindBEAGLE motor imagery training participants imagined the movement of their left or right hand in random order. Children were asked to imagine movement of their right hand to signal a “yes” response and their left hand to indicate “no”.

Controlling a floating cube

An animated cube was presented on the computer screen; using the EPOC+ participants were trained to use motor imagery to push the floating cube away from them or to remain relaxed in order to keep it stationary.

Controlling a remote-controlled car

Using the EMOTIV software, the “push” training was mapped to a keyboard input. A custom made script sent the keyboard command via serial port to a custom interface which was connected to the computer via USB cable. A commercially available toy car was modified to be controlled by this interface. Participants attempted driving the car forward and stopping it at a target location on command as previously described [11]. EMG signals from eyebrow raising could be used to reverse the car.

Sphero SPRK+ control

**Figure 3:**
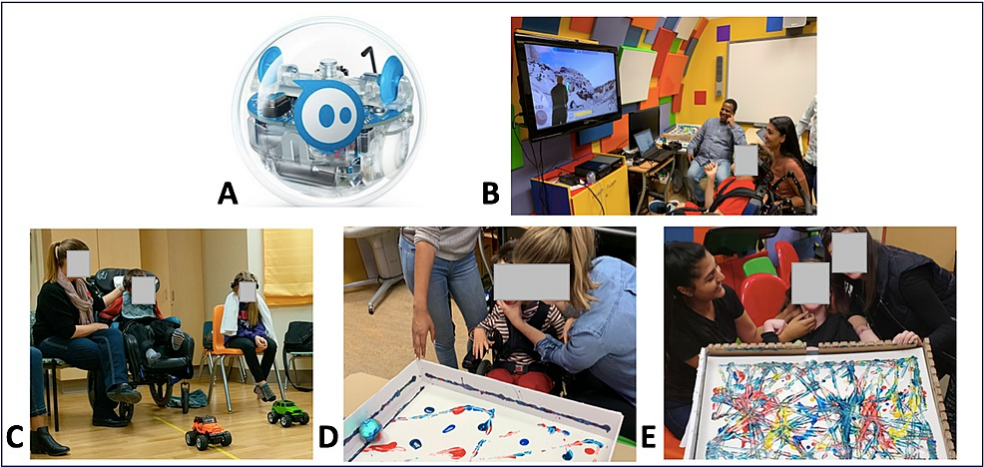
BCI Activities and Clinical Applications. (A) Sphero SPRK+ robot used to create brain-computer interface (BCI) paintings (B) A young man proficient in BCI gaming tries a new popular commercial game modified for EMOTIV control. (C) A young boy and his sister race remote control cars using the EMOTIV. (D) (E) Sphero painting activity. Participants wearing the EMOTIV EPOC+ headset are seated facing a canvas. The Sphero SPRK+ is placed within a bordered canvas with blobs of paint and colours chosen by the participant who then drives the Sphero SPRK+ to create the painting.

A Sphero SPRK+ robot was controlled via keyboard input from the EMOTIV software. The “push” command was used to move the spherical robot in a forward direction using Bluetooth connectivity (Figure 3A). Rotation of the robot to change direction was controlled by either a manual keypress by the experimenter or EMG recorded from eyebrow raising. The second activity allowed participants to drive the robot over a canvas covered in paint creating unique artwork (see Figure 3D, 3E).

iPad control

Participants attempted navigating through icons on an iPad using the EPOC+ to open a smart home application to turn on a desk lap or fan by activating a smart switch. Commands were recorded, mapped and used to control a custom interface in a similar manner as in the car task. A commercially available iPad keyboard was modified to be controlled by this interface. 

Computer games

A suite of BCI-driven computer games was developed including premade and available online as well as custom-designed. All games were played using a single command generated by the user thinking of pushing the character or item on the screen. Push commands were mapped to a keyboard input sent directly to the game. Examples of games include using the push command to have the onscreen character jump over obstacles and target shooting.

Spelling

**Figure 4:**
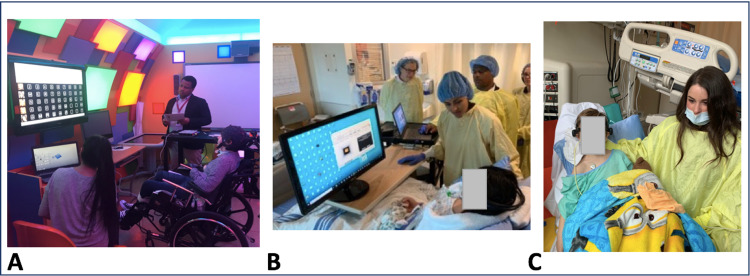
BCI Activities and Clinical Applications Continued (A) An adolescent attempts to use the g.tec intendiX P300-based speller. (B) Two children with acute locked-in syndrome secondary to brainstem stroke and (C) acute necrotizing encephalopathy were able to demonstrate their full consciousness and engage in multiple brain-computer interface (BCI) activities.

The intendiX system included a communication board on a computer screen (Figure 4A) and participants used a visual P300 paradigm to select letters. A classifier was generated after a 5-minute training session. During spelling, participants were asked to attempt to select letters by counting the number of times their target letter flashed on the screen.

Yes/No communication

The mindBEAGLE system used vibro-tactile and auditory evoked potentials and motor imagery paradigms for “yes”/”no” communication and to assess consciousness.

## Results

Participants

At the time of this report, the referral process has identified >30 potential candidates of which 10 have been recruited. All families approached accepted the invitation to join. Participant characteristics and BCI experience are summarized in Table 3. Two adults with locked-in syndrome secondary to brainstem stroke also participated in a modified program but are not described here.

**Table 3:** Participant Demographics. Information on participant pathology and outcomes along with time spent using brain-computer interface (BCI) and paradigms attempted. ANEC: Acute Necrotizing Encephalopathy of Childhood, NAIS: Neonatal Arterial Ischemic Stroke, GBS: Guillain-Barré Syndrome, QCP: Quadriplegic Cerebral Palsy, MI: Motor Imagery, VEP: Visual Evoked Potential, AEP: Auditory Evoked Potential, VTP: Vibrotactile Evoked Potential

Parti.	Sex	Age (yrs)	Pathology	Motor syndrome	Verbal Comm	Epilepsy	BCI Use Time (hrs)	Paradigms Attempted
P1	M	11	Extreme prematurity	Dyskinetic QCP	Y	N	122	MI, VEP
P2	M	12	Lesch-Nyhan Disease	Dyskinetic QCP	N	N	40	MI, VEP
P3	F	14	Bilateral stroke	Mixed QCP, Non-verbal	N	Y	73	MI, VEP, VTP
P4	F	16	Brainstem stroke	Locked-in Syndrome	N	Y	7	MI, VEP, VTP, AEP
P5	M	6	ANEC	Acute Brainstem Dysfunction	N	N	5	MI
P6	M	11	Kernicterus	Dyskinetic QCP	N	N	55	MI, VEP
P7	M	8	Bilateral Schizencephaly	QCP	N	Y	18	MI
P8	F	13	Brainstem Injury	Acute Brainstem Dysfunction	N	N	10	MI, VEP
P9	M	8	NAIS, GBS	Dyskinetic QCP	N	N	4	MI, VEP
P10	F	7	Bilateral stroke	Mixed QCP	N	Y	1	MI

A total of 334 hours of BCI sessions were well tolerated with no serious adverse events. Sessions lasted approximately 60 minutes though some continued for up to two hours. Parents often reported child fatigue after long sessions. Children were constantly consulted on their preferred activities and desired session length. Most were able to indicate “yes” or “no” responses with parents’ assistance. With the exception of P4, no participants have withdrawn from the program and all expressed interest in continued involvement. Details of each participant’s experience are summarized as follows.

Participant 1

P1 is an 11-year-old male with dystonic quadriplegic CP secondary to extreme prematurity with typical intellectual functioning. He has been enrolled in the BCI program since its inception (three years), attending regular weekly sessions. Challenges have included his wheelchair headrest needing to be removed to prevent interference with the EPOC+ headset. He has been successful in using the motor imagery paradigm to perform all EPOC+ activities. While he also demonstrated the ability to use the intendiX system to spell multiple words, it was difficult to maintain his engagement during this task. BCI has provided him his first opportunity to engage in independent play with his brothers. During his recovery after major surgeries, he has used BCI as an inpatient. We have begun successful home-based BCI use by lending equipment to the family and empowering them to set up and control the system so he can play his favorite BCI games at home.

Participant 2

P2 is a 12-year-old male with severe dystonic quadriplegic CP secondary to Lesch-Nyhan disease, a rare genetic inborn error of metabolism. He has mild developmental delays but is intellectually capable. He has been enrolled in the BCI program for three years successfully demonstrating voluntary control using the EPOC+ for the following tasks; 1) floating cube, 2) toy car racing, 3) Sphero maze and painting and 4) computer games. He was unsuccessful on several attempts using the intendiX speller where he was visibly distracted during each attempt to create a classifier, was unable to maintain gaze on the speller board, and expressed discomfort using the EEG caps (Figure 2A), preferring the EPOC+ headset (Figure 1A). The participant had a love of remote controlled cars and the BCI program gave him his first opportunity to race independently. He has also raced cars against his sisters and parents, who were also using the EPOC+ (Figure 3C). He and his family are interested in adding new commands for multiple degrees of control for the car. EMG activity from eyebrow raise has not provided a reliable additional command.

Participant 3

P3 is a 14-year-old female who suffered bilateral arterial strokes in utero resulting in predominantly spastic quadriplegic CP. She is an intelligent young girl who reads above grade level but has no verbal communication or functional hand use. She is able to signal “yes” by sticking out her tongue. She has been actively engaged in the program for two years attending weekly BCI training sessions. She has been successful using all EPOC+ based tasks including using an iPad to turn on a fan. She had difficulties using the indendiX spelling system and was visibly distracted often diverting her gaze from the computer screen during training. She has developed a passion for art and was previously unable to paint independently, she has now created dozens of unique pieces of BCI artwork (Figure 3E). She has donated some of her work and sold several pieces. One goal is to create more degrees of control for her Sphero painting as she has been unable to use EMG to control directional movement of the robot.

Participant 4

P4 is a 16-year-old female who sustained severe brainstem and thalamic injuries due to hemorrhagic stroke following surgery for a brainstem tumor. There was clinical concern that she had been locked in for approximately three years at the time of her first BCI appointment. Her family reached out to our program after having years of unsuccessful attempts at utilizing eye gaze systems for communication. The participant and her family attended three sessions to explore BCI as a possible tool for communication. She was unable to demonstrate reliable and consistent voluntary control during both the floating cube and toy car racing tasks. Focus was therefore transferred to g.tec mindBEAGLE tasks but she did not demonstrate success across any of the paradigms. Although her previous EEG studies post-injury had been nearly normal, we repeated a clinical study and discovered that she evolved an epileptic encephalopathy with almost continuous generalized spike and wave discharges sometime in the preceding three years. With the help of her treating epileptologists, aggressive treatment was attempted over many months but was unsuccessful. After discussion with the family, her participation in the BCI program was discontinued. 

Participant 5

P5 is a previously healthy six-year-old boy who developed acute necrotizing encephalopathy after contracting influenza. He had acute, bilateral brainstem lesions and a clinical locked-in syndrome. He was unable to move his limbs or communicate verbally and had limited preservation of some eye movements. His parents and intensive care doctors expressed concern about his cognitive awareness and capability. Our BCI team was consulted via the neurocritical care service. The EPOC+ system was brought to the patient’s bedside in keeping with infection control and other hospital best practices. He directed his gaze toward the computer screen and held it there throughout training and could consistently push the cube and keep it stationary on command. Subsequently, he demonstrated success playing an animated computer game after brief training. His family was thrilled to witness his ability to voluntarily control these activities while confirmation of his consciousness informed his care planning in the Pediatric Intensive Care Unit (PICU). During later sessions he demonstrated successful control in driving the toy car to designated targets. Even after regaining arm movement and verbal communication he continued using BCI as a recreational activity during his hospital admission. He created a painting using the BCI and Sphero which still hangs in his home today. He has since made a full recovery.

Participant 6

P6 is an 11-year-old male with dyskinetic quadriplegic CP secondary to kernicterus who has been enrolled in the BCI program for 18 months. He has typical intellectual function, good vision and hearing but no functional hand use. While he has no verbal communication, he is able to raise and lower his gaze to signal “yes” and “no” responses, respectively. He and his family have been attending weekly BCI sessions since his enrollment 18 months ago and are interested in continuing involvement. He has extremely limited control of his neck and during EPOC+ activities he required a parent to hold his head in position. He has been successful in using each of the BCI paradigms attempted (see Table 2) and has expressed a preference for painting with the Sphero. He was also able to accurately select five out of five target letters from the spelling board and expressed that he would be willing to train further using the intendiX speller. 

Participant 7

P7 is an eight-year-old male with quadriplegic CP secondary to bilateral schizencephaly. This young boy appears to be intellectually typical with good vision but no functional use of his extremities. He is non-verbal but capable of expressing “yes” and “no” through changes in facial expression, which his parents assist in interpreting. This participant has attended weekly BCI sessions for six months and has demonstrated good voluntary control during all EPOC+ tasks. He has a preference for Sphero painting as well as a new custom-designed target shooting computer game. We chose not to pursue the intendiX task with this participant because he found the headset uncomfortable. He is excited to bring other family members along to his BCI sessions to showcase his ability. During his participation in the program he has had his first opportunity to create tangible and independent artwork and was excited to play video games that resembled the ones his dad plays at home. 

Participant 8

P8 is a previously healthy 13-year-old female who had an acute brainstem stroke secondary to traumatic vertebral artery dissection. She was intubated in a locked-in state days later with no ability to communicate. Her intensive care team was concerned she was not conscious. She was referred to the BCI program by her neurocritical care neurologist. The EPOC+ system was brought to the bedside in accordance with infection control policies (Figure 4B). After a 10-minute training period, she could push the cube and hold it in position on command. She successfully attempted an EPOC+-based computer game, making her character jump over obstacles. This initial success was encouraging to her family and convinced the hospital staff of her intact consciousness. Training continued for several weeks where she was successful at Sphero painting but unable to use the intendiX communication board. While intubated in the PICU, headset placement was often difficult, requiring pillows or others to physically support her head. We attempted to introduce a second “left” command using left hand squeeze imagery-related SMR pattern during which she physically squeezed her hand for the first time. Over months she regained motor and communication skills and no longer required BCI systems.

Participant 9

P9 is an eight-year-old male with dyskinetic quadriplegic CP secondary to bilateral, deep neonatal arterial ischemic strokes secondary to group B streptococcal meningitis. He has very little functional hand use and no verbal communication but is able to sign “yes” and “no” and is probably intellectually normal. He has completed three introductory BCI sessions and demonstrated success in many EPOC+ tasks including floating cube, toy car racing, single command computer games, Sphero maze and painting. During training, his wheelchair headrest is removed and he can maintain his head position independently. He was unsuccessful using the intendiX and became visibly distracted after three minutes of training. He was able to select letters in close proximity to the target letter. His mother suggested that this may be difficult because he uses an eye gaze system which requires less sustained attention. His excessive movements created seating challenges and he was moved to a bean bag style chair which allowed him to relax and continue training.

Participant 10

P10 is a seven-year-old female with quadriplegic CP secondary to fetal strokes occupying most of the middle cerebral artery territories bilaterally. She is a bright and social young girl who has no functional use of her extremities. She is non-verbal but can express some yes and no responses through changes in facial expression which her parents help to interpret. She has completed her initial screening session and has demonstrated voluntary control of the floating cube, toy car and EPOC+ based computer games. Despite living outside of the city, this participant and her family have expressed interest in attending monthly sessions and we are also pursuing remote or home-based options.

**Figure 5:**
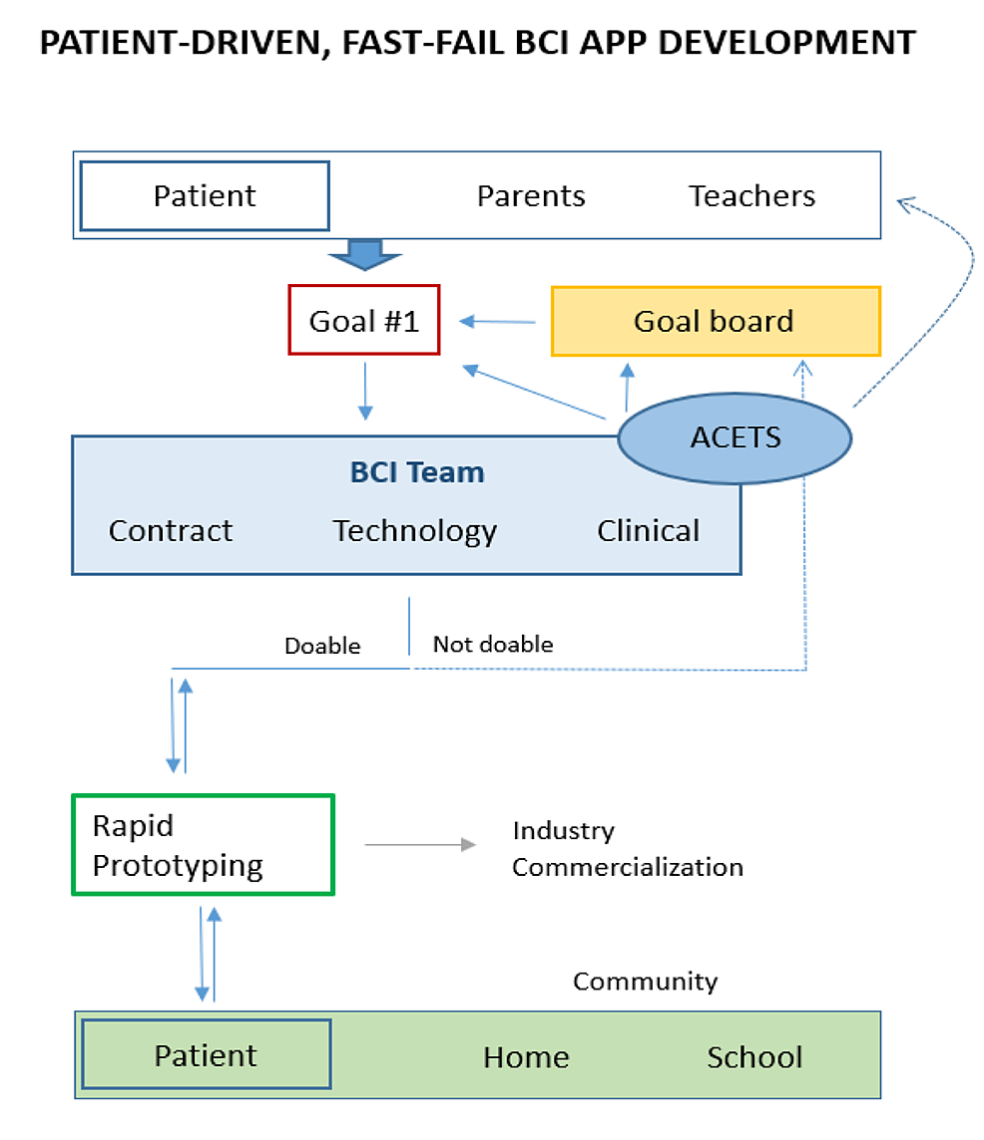
Fast-fail, personalized goal-setting. A new program model designed to integrate parent, teacher and participant objectives for brain-computer interface (BCI) control. Attainable goals for BCI applications will be set with the family, BCI team and health care professionals familiar with assistive technology (ACETS: Augmentative Communication and Educational Technology). As the child continues to attend regular BCI training sessions, rapid prototyping will take place to develop new applications. These will ultimately be brought back to the child for pilot testing and further developed. Eventual transition of applications into the home, school and for commercialization are important considerations moving forward.

Based on the above experience, including input from users and their families, we have developed a personalized, goal-oriented, “fast-fail” approach to drive the next phase of program development (Figure 5).

## Discussion

We report the early establishment of a clinical BCI program for children with severe disability. Systematic screening identified many potential candidates. Engagement from patients and their families was high with 100% enrolment, keen participation, and ongoing commitment. Nearly all participants succeeded in acquiring BCI control and many developed diverse skills and realized personal accomplishments that were not previously possible. Factors related to technologies, goals, and individual differences of each participant and family were identified to facilitate program growth.

We believe the high level of engagement and commitment from our young participants and their families attests to the potential value of this clinical BCI program. Long-term engagement occurred for all but one, suggesting some degree of benefit gained through participation. This is encouraging considering our candor with participants and their families avoiding any unrealistic expectations. Canadian specialists who care for persons with severe disability have confirmed a high level of potential clinical utility [10]. We recruited a critical mass of well-qualified participants from a moderately sized, single referral center. It seems clear that there is an unmet need for advanced assistive technology in severely disabled children and, consistent with international policy on the rights of persons with disability, we would advocate for the further growth of pediatric BCI programs. 

Meaningful engagement requires motivation, which was a challenge for multiple users. Adult studies have described the impact of motivation on BCI performance and this may be exacerbated in young children [19]. Several participants reported high interest and success using motor imagery for play but then lacked engagement during the P300 spelling task. This could be related to the engaging feedback and short training of motor imagery activities in contrast to spelling tasks. Effective feedback is especially necessary when attempting BCI tasks for the first time [20]. Performance using the P300 speller relies on the quality of the classifier produced; a poorly trained classifier hinders performance and may reinforce poor strategy [20]. Studies in adults suggest feedback should be supportive and dynamic, providing information on how to improve [21]. This could be helpful for young children during tasks that may not have intuitive outcomes. For example, it may be clearer to the child to focus on “pushing” to move a toy as opposed to counting flashing letters to spell. Children need to participate in frequent practice to improve BCI skills [20]. Difficulties adopting assistive technology for children with severe disability are related to limited adaptability of software for different needs [22]. Adaptable and engaging tasks are likely to positively impact BCI performance for these children.

One solution to enhance motivation and attention in young BCI users may be gaming. The range of simple BCI games in our program, while popular, were quite limited and participants were often quick to reach competency. Gamification of BCI tasks has the potential to improve engagement and motivation and serve as a tool to train specific tasks [23,24]. Research in children with attention deficit hyperactivity disorder often utilizes game-based training to maintain attention [25]. That games should differ in content and theme and have varying levels of difficulty needs to be applied to BCI [20]. Participants indicated keen interest in the commercially available games their friends and siblings play. Connecting with leading game developers to advance BCI-specific applications can facilitate skill training, higher levels of recreational therapy, and social networking (e.g. online gaming networks). Parents also expressed interest in educational applications and advancing engaging tasks for spelling, reading, math and other subjects is an intended aim of our program.

Important challenges were identified. Participant 4 was unable to use the BCI likely due to underlying pathological EEG activity. Clinical data including baseline EEG may help inform participant selection. Neuroimaging was used to help estimate cognitive potential in the face of severe physical disability. Most had relative sparing of the cortex, the source of EEG signal and key to intellectual functioning. While mechanisms of BCI control in CP have not been well studied, the health of the cortex is almost surely related to BCI performance. However, the cortex may not need to be entirely intact including several participants here and previous studies of children with perinatal stroke can demonstrate good BCI performance [12]. This selection factor may have been overlooked in previous studies of BCI users with more generic “cerebral palsy” and our possibly high success rate may in part reflect this anatomical screening approach.

Another important consideration is EEG headset design for such complex pediatric populations. Children with severe physical disability often have limited head and neck control and/or severe dyskinetic movements. Accordingly, headsets like the EPOC+ often required an assistant to physically support the participant to maintain electrode placement. This can result in discomfort for the child and assistant while potentially introducing unwanted EEG noise. Clinical EEG caps may overcome this but created additional discomfort in some cases. Innovative approaches to EEG sampling and supportive measures to improve physical comfort, like the beanbag chair example, are required.

We would also highlight the importance of a multidisciplinary team. This starts and ends with affected children and their families whose active engagement is essential to program success. Adoption of assistive technology is reliant on many factors, however a common reason for abandonment is incongruence with the users’ needs [26]. We must also improve awareness and input from health care professionals who are best able to identify eligible children and carry deep understanding of their needs and challenges. BCI development is proceeding rapidly but focusing only on technological advancement without affording equal attention to the needs of children and families will limit progress [19,20].

## Conclusions

Patient-centered, clinical BCI programs appear to be feasible for children with severe neurological disability. We have demonstrated that such children are not only able to use simple BCI systems for play and communication, but they and their families are potentially interested in long-term involvement as members of a clinical BCI program. Children with severe quadriplegic CP and no verbal communication, who had never had the opportunity to participate in activities independently, were able to create artwork, drive remote-controlled cars, play simple single-control computer games and slowly select letters from a communication board. While these were personally and clinically meaningful accomplishments and a step forward in the field of accessible technologies for severely disabled children, there are still many gaps to fill.

As demonstrated, there is a significant lack of BCI research in children with disabilities and currently available systems are not adapted to their unique physiology, needs, or interests. The commercially available systems used in our program had limited flexibility for pediatric use including rigid headset structure and electrode layout. The applications available were also limited and tasks were not always presented in an engaging way for our young participants. Despite these challenges, we have shown that buy-in from participants and their families is high. This motivation to participate in our clinical program highlights the lack of options, steadfast advocacy of parents for their children, and marked opportunities for these families. At the same time it represents a powerful, untapped resource by which the advancement of BCI neurotechnologies can be enhanced to potentially impact independence and quality of life for children whose current options are extremely limited. 

BCI technological progress is rapid and inevitable; matching this progress from the clinical side and the perspective of disabled children and their families is essential for the full potential of pediatric BCI to be realized. We hope our findings will encourage such collaborations, support the development of BCI programs focused on children elsewhere, and one day expand to include more impactful applications such as wheelchair control, fluent communication, increased access to entertainment, and improvement in overall independence for these children. 
